# Oncolytic Herpes Simplex Virus for Glioblastoma: Molecular Engineering, Tumor Microenvironment Barriers, and Clinical Translation

**DOI:** 10.3390/cimb48050499

**Published:** 2026-05-13

**Authors:** Jiayu Liu, Yuxin Wang, Zhao Gao, Tongtan Liu, Ao Xu, Wenxuan Li, Mei Li, Xiaomeng Song, Baorui Guo, Huadong Wang, Wenying Lv, Jianning Zhang

**Affiliations:** 1 Senior Department of Neurosurgery, Chinese PLA General Hospital, Beijing 100853, China; liujiayu@pku.edu.cn (J.L.); wangyuxin037@163.com (Y.W.); gaz3141592654@163.com (Z.G.); xuao818@163.com (A.X.); lwenx1596@163.com (W.L.); 18332758771@163.com (M.L.); axmsong@163.com (X.S.); xian1002022@163.com (B.G.); 2 The Brain Cognition and Brain Disease Institute, Shenzhen-Hong Kong Institute of Brain Science, Shenzhen Institutes of Advanced Technology, Chinese Academy of Sciences, Shenzhen 518055, China; tt.liu2@siat.ac.cn (T.L.); hd.wang@siat.ac.cn (H.W.)

**Keywords:** glioblastoma, oncolytic herpes simplex virus, tumor microenvironment, viro-immunotherapy, molecular engineering, translational therapy

## Abstract

Glioblastoma (GBM) remains the most aggressive primary malignant brain tumor in adults, with limited survival benefit from the current standard of care consisting of maximal safe resection, radiotherapy, and temozolomide-based chemotherapy. The highly infiltrative growth pattern, profound intratumoral heterogeneity, and strongly immunosuppressive tumor microenvironment together contribute to therapeutic resistance and frequent recurrence. In this context, oncolytic herpes simplex virus (oHSV) has emerged as a promising therapeutic platform for glioblastoma because of its dual capacity to directly lyse tumor cells and stimulate antitumor immune responses. In addition, the large viral genome and well-characterized biology of herpes simplex virus enable extensive genetic engineering to improve tumor selectivity, safety, and immunomodulatory function. In this review, we summarize the molecular design strategies that have driven the development of oHSV for glioblastoma, including attenuation of neurovirulence, enhancement of tumor-selective replication, and arming with immune-stimulatory transgenes. We further discuss the major biological barriers within the GBM tumor microenvironment that continue to limit therapeutic efficacy, with particular attention given to representative engineered oHSV platforms and the lessons learned from preclinical and early-phase clinical studies. A dedicated section examines these barriers in detail, including restricted intratumoral viral spread, antiviral innate immunity, and immunosuppressive myeloid cell dominance. We also review current efforts to improve outcomes through rational combination strategies with radiotherapy, immune checkpoint blockade, cytokine modulation, and other multimodal approaches. Although encouraging advances have been achieved, the clinical translation of oHSV therapy for glioblastoma still faces substantial challenges in patient selection, delivery optimization, response assessment, and treatment integration. A deeper understanding of virus–host–tumor interactions and more precise engineering of viral platforms may help unlock the full potential of oHSV-based therapy. Overall, oHSV represents one of the most compelling translational approaches in glioblastoma and provides a valuable framework for the development of mechanism-driven viro-immunotherapy in neuro-oncology.

## 1. Introduction

Glioblastoma (GBM) remains the most aggressive and lethal primary malignant brain tumor in adults. Despite decades of investigation, the current standard of care, consisting of maximal safe resection followed by radiotherapy with concomitant and adjuvant temozolomide, provides only limited survival benefit for most patients [1,2]. Recurrence is nearly universal, and long-term survival remains uncommon [3]. The poor prognosis of GBM reflects not only its highly infiltrative growth pattern, but also its marked spatial and temporal heterogeneity, profound immunosuppressive microenvironment, and remarkable capacity to adapt to therapeutic pressure [4]. Together, these features have made GBM one of the most treatment-resistant solid tumors in clinical oncology. The limited success of conventional cytotoxic therapies and the overall modest benefit achieved with most targeted and immune-based approaches have therefore driven continued interest in innovative treatment platforms capable of overcoming multiple resistance mechanisms simultaneously.

Among these emerging strategies, oncolytic virotherapy has attracted sustained attention as a biologically versatile and mechanistically distinct therapeutic approach [5]. In contrast to conventional anticancer modalities that rely primarily on direct cytotoxicity or pathway inhibition, oncolytic viruses are designed to selectively infect, replicate within, and lyse tumor cells while simultaneously reshaping the tumor microenvironment. This dual mechanism is particularly appealing in GBM, where effective therapy likely requires not only tumor cell killing, but also reversal of local immune suppression and induction of durable antitumor immunity [6]. Oncolytic virotherapy thus offers a conceptual bridge between direct tumor debulking and cancer immunotherapy, making it especially relevant in a disease that is both locally destructive and immunologically complex.

Among the various viral platforms under investigation, oncolytic herpes simplex virus (oHSV) has emerged as one of the most extensively studied and clinically advanced candidates for GBM [7]. Several features make herpes simplex virus an attractive backbone for therapeutic engineering. Its large double-stranded DNA genome provides substantial capacity for genetic modification and transgene insertion [8]. Its biology has been well characterized over decades of virological and neurobiological research, enabling rational manipulation of neurovirulence, replication competence, and tumor selectivity. In addition, the availability of antiviral agents offers an added layer of clinical controllability that is particularly relevant for intracranial application [9]. These properties have supported the development of multiple generations of engineered oHSV constructs designed to improve safety, tumor selectivity, intratumoral replication, and immune stimulation [10,11].

The rationale for applying oHSV to GBM is strong, but the path to meaningful clinical benefit has proven more complex than initially anticipated. Preclinical studies have repeatedly demonstrated that oHSV can mediate potent antitumor effects through direct oncolysis, enhancement of immunogenic cell death, release of tumor-associated antigens, recruitment of immune effector cells, and remodeling of the tumor immune milieu. However, these encouraging mechanisms do not automatically translate into durable efficacy in patients. The GBM microenvironment presents multiple barriers to successful virotherapy, including restricted viral dispersion, innate antiviral responses, abundant tumor-associated macrophages and microglia with predominantly immunosuppressive functions, extensive inter- and intratumoral heterogeneity, and the physical constraints imposed by infiltrative growth and altered extracellular architecture [12]. As a result, although early-phase clinical trials have generally supported the safety and feasibility of oHSV-based therapy, therapeutic efficacy has remained variable and often incomplete [13,14].

This gap between biological promise and clinical impact highlights an important need for a more integrated understanding of oHSV therapy in GBM. A simple descriptive overview of viral platforms is no longer sufficient. Instead, current progress in the field calls for a critical synthesis that links viral engineering strategies with tumor biology, immune context, delivery challenges, and translational outcomes. In particular, it is increasingly clear that the success of oHSV in GBM depends not only on the intrinsic lytic potential of the virus, but also on how effectively the viral platform is engineered to function within the unique ecological and immunological constraints of the brain tumor microenvironment. Likewise, the future of oHSV is likely to depend on rational combination strategies, optimized delivery paradigms, improved patient selection, and more precise approaches to response assessment [9].

**Figure 1:**
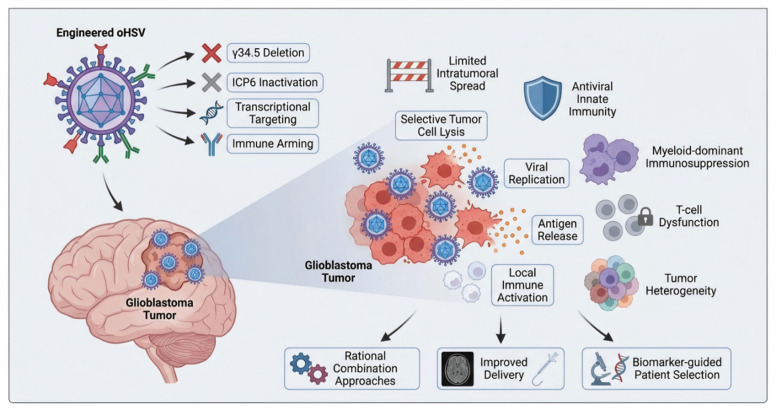
Overview of oncolytic herpes simplex virus (oHSV) therapy in glioblastoma. Engineered oHSV platforms are designed to improve safety, tumor selectivity, and immunostimulatory capacity through strategies such as *γ34.5* deletion, *ICP6* inactivation, transcriptional targeting, and immune arming. These modifications are intended to promote selective tumor cell lysis, viral replication, antigen release, and local immune activation. However, the therapeutic efficacy of oHSV is constrained by the glioblastoma microenvironment, including limited intratumoral spread, antiviral innate immunity, myeloid-dominant immunosuppression, T-cell dysfunction, and tumor heterogeneity. Current optimization strategies therefore focus on rational combination approaches, improved delivery, and biomarker-guided patient selection.

In this review, we discuss the development of oHSV for GBM from a molecular and translational perspective. We first summarize the biological features that make herpes simplex virus a suitable therapeutic platform in neuro-oncology, and then examine the major engineering strategies used to enhance safety, tumor selectivity, and immune activation. We next analyze the key microenvironmental and host-related barriers that continue to limit efficacy in GBM, with particular attention to antiviral immunity, myeloid-driven suppression, and tumor heterogeneity. We also review representative preclinical and clinical studies, highlighting the major lessons learned from currently available oHSV platforms [15,16,17,18,19]. Finally, we discuss emerging combination strategies and future directions that may help unlock the full therapeutic potential of oHSV in GBM. By focusing on the intersection of molecular engineering, tumor biology, and clinical translation, this review aims to clarify why oHSV remains one of the most promising yet still incompletely realized therapeutic approaches in GBM (Figure 1).

## 2. Why oHSV Is a Suitable Platform for GBM Therapy

Among the currently available oncolytic virus platforms, oHSV has emerged as one of the most attractive candidates for GBM therapy because it combines a highly engineerable viral backbone with biologically relevant antitumor mechanisms [9]. In principle, an ideal oncolytic platform for GBM should satisfy several requirements simultaneously: it should permit selective replication within tumor cells, allow precise attenuation of pathogenicity in normal brain tissue, retain sufficient payload capacity for therapeutic transgenes, and generate both direct cytolytic and immune-mediated antitumor effects. oHSV fulfills these requirements more comprehensively than many other viral systems currently under investigation, which explains its prominent position in both preclinical and clinical neuro-oncology research.

A major advantage of herpes simplex virus lies in the structure of its genome. Herpes simplex virus (HSV) is a large double-stranded deoxyriboNucleic acid (DNA) virus with substantial genomic capacity, which provides broad flexibility for therapeutic engineering. This feature enables investigators to delete virulence-associated genes, introduce tumor-selective regulatory elements, and insert immunomodulatory or therapeutic transgenes without completely compromising viral fitness. In contrast to smaller viral platforms with more limited packaging space, oHSV can be redesigned in a modular manner to enhance safety, tumor specificity, and immunostimulatory potential at the same time. This large genomic “engineering space” has been a central factor in the development of successive generations of oHSV constructs for GBM, ranging from early attenuated viruses to more sophisticated armed and transcriptionally targeted vectors [20].

Equally important, the biology of HSV has been extensively characterized through decades of virological and neurobiological investigation. Because the molecular determinants of viral neurovirulence, replication, host interaction, and latency have been studied in detail, HSV offers a level of rational manipulability that is especially valuable for intracranial application. This is a critical consideration in GBM, where therapeutic strategies must balance efficacy against the risk of damage to surrounding normal brain tissue. The development of oHSV has therefore benefited from a uniquely mature knowledge base, allowing investigators to attenuate neurotoxicity while preserving antitumor activity. In practical terms, this has enabled the design of vectors with reduced pathogenicity in normal cells but retained replication potential in malignant glioma cells, a property that is fundamental to their translational appeal.

Another key strength of oHSV is its dual mechanism of action. Like other oncolytic viruses, oHSV can directly infect susceptible tumor cells, replicate intracellularly, and induce cell lysis. However, the therapeutic effect of oHSV is not limited to direct cytotoxicity. Viral infection can also promote immunogenic cell death, release tumor-associated antigens and danger signals, and stimulate innate and adaptive immune responses within the tumor microenvironment. This feature is especially relevant in GBM, a disease characterized by profound local immunosuppression and poor responsiveness to many conventional immunotherapies [7]. In this setting, oHSV may serve not only as a tumor-lytic agent but also as a biological trigger for in situ immune activation. This capacity to bridge virotherapy and immunotherapy is one of the principal reasons why oHSV continues to attract attention in GBM research.

The neural tropism of HSV also contributes to its relevance in brain tumor therapy. Although native neurotropism raises obvious safety concerns in its wild-type form, it also means that HSV-based vectors are built upon a viral platform inherently capable of interacting with cells and tissues in the central nervous system. Once appropriately attenuated, this feature can be redirected toward therapeutic purposes. In GBM, where local delivery into the resection cavity or intratumoral region is often feasible, the use of an engineered HSV backbone offers practical compatibility with neurosurgical treatment paradigms [21]. This compatibility has supported the development of intratumoral and perioperative administration strategies that are less dependent on systemic delivery than many other anticancer agents.

An additional translational advantage of oHSV is clinical controllability. Unlike some investigational viral platforms for which rescue options are limited, HSV-based therapy benefits from the existence of clinically established antiviral drugs, such as acyclovir and related agents. Although these agents are not intended to eliminate therapeutic activity under routine treatment conditions, their availability provides an additional safety safeguard in the event of unexpected viral toxicity or off-target replication. This controllability has been an important argument supporting the clinical development of oHSV in the central nervous system, where safety considerations remain especially stringent. The presence of an antiviral “off-switch” does not remove all risks, but it does contribute to a more favorable translational profile compared with less easily controlled viral systems.

From a disease-specific perspective, GBM itself presents several features that make it a logical target for oHSV-based therapy. First, the marked resistance of GBM to standard treatment creates a strong clinical rationale for biologically distinct therapeutic approaches. Second, the local recurrence pattern of GBM supports direct intratumoral or intracavitary intervention. Third, the highly immunosuppressive microenvironment of GBM may paradoxically provide an opportunity for oHSV, because viral infection can convert immunologically “cold” tumor regions into more inflamed and antigenically visible lesions. Finally, the adaptability of oHSV allows it to be combined with radiotherapy, immune checkpoint blockade, cytokine expression strategies, and other multimodal interventions, making it an attractive backbone for combination therapy rather than a purely standalone agent [22].

These biological and translational advantages help explain why oHSV has remained one of the most advanced virotherapeutic strategies in GBM. Nevertheless, the suitability of oHSV as a platform does not guarantee clinical success. The same features that make GBM a compelling target—its heterogeneity, local invasiveness, and complex immune ecology—also create major barriers to effective viral spread and durable tumor control. For this reason, the promise of oHSV in GBM depends not only on the inherent strengths of the HSV platform, but also on how precisely the virus can be engineered to function within the unique constraints of the GBM microenvironment. This rationale for precision engineering forms the basis for the next phase of oHSV development and is discussed in detail in the following section.

## 3. Molecular Engineering Strategies of oHSV for GBM

The therapeutic potential of oHSV in GBM depends not only on the intrinsic biology of the viral backbone, but also on how precisely the virus is engineered to balance safety, tumor selectivity, replicative fitness, and immunostimulatory capacity. Because wild-type herpes simplex virus type 1 is naturally neurotropic and neurovirulent, its direct application in the central nervous system is not clinically acceptable. The development of oHSV for GBM has therefore been driven by a central engineering challenge: to attenuate pathogenicity in normal brain tissue while preserving, or even enhancing, antitumor activity in malignant glioma cells. Over the past three decades, this challenge has led to the emergence of several complementary design strategies, including deletion of neurovirulence-associated genes, disruption of viral genes required for replication in normal cells but dispensable in proliferating tumor cells, transcriptional targeting of viral replication, and insertion of therapeutic transgenes that amplify immune activation or tumor killing. Together, these approaches have transformed HSV from a pathogenic neurotropic virus into a highly adaptable therapeutic platform for neuro-oncology.

### 3.1. Attenuation of Neurovirulence and Safety Optimization

One of the earliest and most important advances in oHSV development was the identification of the viral *γ34.5* gene as a key determinant of neurovirulence [23]. Deletion of both copies of *γ34.5* markedly reduces the ability of HSV-1 to cause encephalitis while preserving the possibility of replication in certain tumor contexts. This discovery provided the conceptual foundation for first-generation oHSV vectors designed for intracranial use [24]. In GBM, the biological rationale is straightforward: tumor cells often exhibit altered antiviral defenses, dysregulated stress-response pathways, and permissive metabolic states that can partially compensate for viral attenuation, whereas normal neurons and glia are far less supportive of replication by *γ34.5*-deleted viruses. As a result, *γ34.5* deletion became a foundational strategy for increasing central nervous system (CNS) safety while retaining tumor-directed activity.

A related safety strategy involved disrupting additional viral genes linked to efficient replication in non-dividing normal cells. Among these, *ICP6*, which encodes the large subunit of viral ribonucleotide reductase, became particularly important. Because actively proliferating tumor cells provide a more favorable nucleotide environment than quiescent normal cells, *ICP6*-deficient viruses are relatively impaired in normal tissues but can remain replication competent in malignant glioma cells. The combination of *γ34.5* deletion and *ICP6* inactivation became a classic design logic in oHSV development and was embodied in G207, one of the best known and most extensively studied first-generation constructs. G207 contains deletions in both *γ34.5* loci and a lacZ insertion that disrupts *UL39/ICP6*, producing a virus with attenuated neurovirulence yet preserved replication in dividing tumor cells. This construct played a major role in establishing the clinical feasibility of oHSV therapy for malignant glioma [25].

Although safety attenuation is essential, excessive attenuation can compromise therapeutic potency. This trade-off has been one of the defining tensions in oHSV engineering. First-generation vectors achieved a favorable safety profile, but in some contexts their replication capacity and intratumoral spread were limited, especially in the hostile microenvironment of GBM. This recognition motivated the development of second-generation vectors designed not simply to further attenuate the virus, but to restore antitumor fitness in a controlled and tumor-restricted manner. In other words, the goal shifted from “making HSV safe” to “making HSV selectively powerful in tumor cells while remaining safe in the normal brain.”

### 3.2. Tumor-Selective Replication Strategies

After the success of early attenuation approaches, the next major engineering objective was to improve tumor selectivity without sacrificing replicative efficiency. One strategy involved transcriptional targeting, in which essential viral genes are placed under the control of tumor-associated promoters so that replication is preferentially supported in cancer cells rather than normal tissue. In GBM, *nestin* has been a particularly attractive regulatory target because it is highly expressed in glioma cells and glioma stem-like populations but not broadly active in the mature normal brain. This logic underlies vectors such as rQNestin34.5v.2 and the related clinical construct CAN-3110, in which the previously deleted y34.5 gene is conditionally reintroduced under control of the *nestin* promoter. This design attempts to solve a central problem of first-generation oHSV vectors: how to recover the replication benefits of *γ34.5* expression while restricting them to tumor cells.

This transcriptional retargeting strategy reflects a broader shift in the field from static attenuation to conditional competence. Rather than simply deleting virulence factors, newer vectors attempt to restore selected viral functions only in the molecular context of the tumor. In GBM, where intratumoral spread is often a major limitation, this conditional restoration of replicative capacity is particularly attractive. It offers a way to improve viral amplification in *nestin*-positive tumor regions while maintaining safety constraints in surrounding normal tissue. The clinical relevance of this concept has been strengthened by the first-in-human trial of CAN-3110, which linked intratumoral oHSV treatment to immunologic activation and suggested survival associations in subsets of recurrent GBM patients. Although these findings do not yet establish definitive clinical efficacy, they provide important proof that precision viral engineering can reshape both viral behavior and host response in human GBM [19].

Another approach to improving tumor-selective replication has been to modify viral interactions with cellular antiviral pathways. GBM cells often harbor defects in interferon signaling, translational control, and antiviral stress responses, which can be exploited to create viruses that remain restricted in normal tissue but replicate more efficiently in tumor. Chimeric constructs such as C134 were developed in part to address the replication limitations of highly attenuated vectors by inserting heterologous elements that help counteract host antiviral shutdown pathways. C134 includes the human cytomegalovirus (HCMV) *IRS1* gene, which enhances late viral protein synthesis in the setting of protein kinase R (PKR) activation, thereby improving replication relative to some earlier *γ34.5*-deleted viruses while remaining substantially attenuated compared with wild-type HSV. This strategy illustrates a more sophisticated phase of oHSV design in which viral engineering is directed not only at tumor targeting, but also at selective resistance to host antiviral restriction mechanisms within the tumor environment [11,26].

### 3.3. Arming oHSV with Immunostimulatory Transgenes

As the field increasingly recognized that direct oncolysis alone might be insufficient for durable control of GBM, viral engineering began to incorporate immune-stimulatory payloads. This led to the development of “armed” oHSV constructs that function not only as tumor-selective replicating agents but also as local gene delivery vectors. The rationale is especially compelling in GBM, where systemic immunotherapy often underperforms because of a profoundly suppressive tumor microenvironment and limited immune cell activation. By expressing immune-modulating molecules directly within the tumor, armed oHSV vectors aim to convert local viral infection into a broader antitumor immune event.

Interleukin-12 (IL-12) has emerged as one of the most important transgenes in this context. IL-12 can enhance cytotoxic T-cell and natural killer cell function, promote interferon-γ production, and help shift the tumor microenvironment toward a more pro-inflammatory state. M032, a second-generation *γ34.5*-deleted oHSV expressing human IL-12, represents one of the most clinically relevant examples of this strategy. The design of M032 reflects the evolving therapeutic ambition of oHSV engineering: rather than treating the virus purely as a lytic agent, the vector is used to integrate direct tumor destruction with localized immune modulation. Preclinical and translational work supporting M032 has emphasized not only its oncolytic properties but also its potential to stimulate an antitumor immune response that extends beyond the cells directly infected by the virus [17].

The arming strategy also has broader implications. It allows oHSV to serve as a platform for rational combinatorial biology within a single agent. A transgene can be chosen to promote antigen presentation, enhance effector-cell recruitment, counteract local immunosuppression, or synergize with checkpoint blockade and radiotherapy. In principle, this approach is highly suitable for GBM, where failure often results not simply from insufficient cytotoxicity, but from the inability to establish a durable and coordinated immune response. Thus, immune-armed oHSV vectors represent a transition from first-generation oncolytic design toward true viro-immunotherapy [5].

### 3.4. Representative Engineered oHSV Platforms in GBM

**Figure 2:**
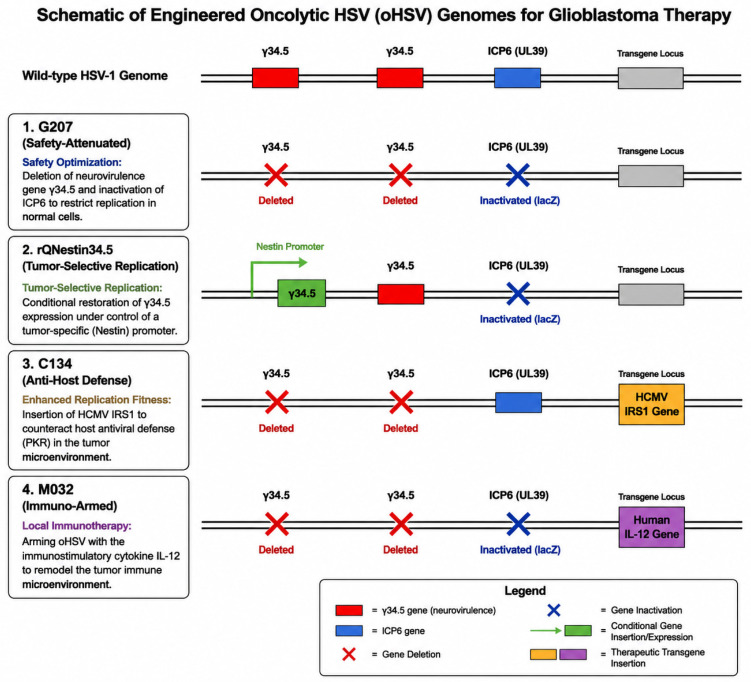
Schematic illustration of genetic engineering strategies for oHSV in glioblastoma therapy. This figure illustrates the key genomic modifications from the wild-type HSV-1 genome to representative engineered oHSV constructs (G207, C134, M032, and CAN-3110). The text boxes on the left summarize the core objectives of each generation of engineering, while the linear genome maps on the right visually depict the specific mutation sites. Key modifications include: deletion of the neurovirulence gene *γ34.5* and inactivation of *ICP6* in G207 for safety attenuation; insertion of the hCMV *IRS1* gene in C134 to enhance replicative fitness in the tumor microenvironment; arming with the human IL-12 gene in M032 for local immunotherapy; and conditional restoration of *γ34.5* expression under the tumor-specific *Nestin* promoter in CAN-3110 for transcriptional targeting. This schematic systematically outlines the molecular design evolution of oHSV in terms of safety, replication efficiency, and immunomodulation.

Several engineered oHSV platforms collectively illustrate the major stages of development in this field. As outlined in Section 3.1, the first-generation oHSV G207 emphasized safety primarily through dual *γ34.5* deletion and *ICP6* disruption. Subsequently, building on the foundational *γ34.5* deletion strategy, newer vectors aim to combine the safety benefits of attenuation with a conditional recovery of *γ34.5*-mediated replication. A schematic illustration of these genetic engineering strategies for oHSV is shown in Figure 2. Rather than re-describing each vector in detail here, their key genetic features, design logic, and translational status are summarized in Table 1. Taken together, these representative platforms show how oHSV engineering in GBM has evolved from simple neurovirulence reduction toward more functionally integrated viro-immunotherapy strategies.

### 3.5. Summary of Engineering Logic

Taken together, the history of oHSV development in GBM can be understood as a progression through three broad engineering stages. The first stage focused on safety, mainly through deletion of neurovirulence-associated genes. The second stage sought to recover tumor-selective replication and improve intratumoral fitness through conditional targeting and antiviral-pathway modulation. The third stage expanded the role of oHSV from a lytic vector to an immunotherapeutic platform by incorporating transgenes such as IL-12 and by designing viruses capable of reshaping the tumor microenvironment. These stages are not mutually exclusive; rather, they now converge in the design of modern oHSV platforms for GBM. The central challenge moving forward is to integrate these design principles in a way that achieves sufficient replication, selective tumor killing, and durable immune activation without compromising CNS safety (Table 1).

## 4. The GBM Microenvironment as a Barrier to oHSV Therapy

**Figure 3:**
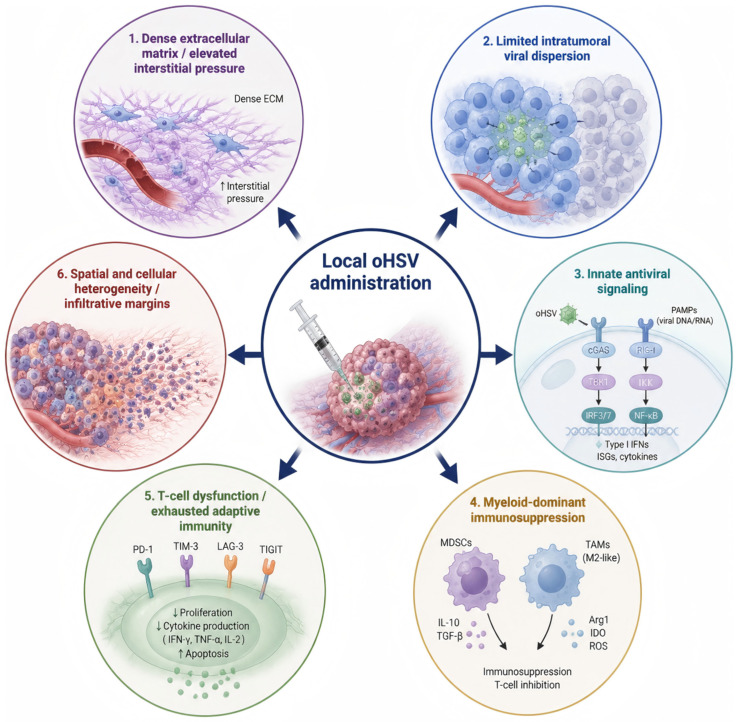
Biological and physical barriers limiting oHSV therapy in the glioblastoma tumor microenvironment. Local oncolytic herpes simplex virus (oHSV) administration in glioblastoma faces six key barriers: (1) Dense extracellular matrix (ECM) and elevated interstitial pressure, which physically restrict virus spread; (2) Limited intratumoral viral dispersion, reducing therapeutic reach; (3) Innate antiviral signaling (e.g., cGAS-STING, RIG-I-MAVS pathways) triggering type I interferons (IFNs) and interferon-stimulated genes (ISGs) that suppress viral replication; (4) Myeloid-dominant immunosuppression (via MDSCs, TAMs/M2-like macrophages) secreting IL-10, TGF-β, IDO, ROS to inhibit T-cell function; (5) T-cell dysfunction/exhausted adaptive immunity (marked by PD-1, TIM-3, LAG-3, TIGIT upregulation) with reduced proliferation, cytokine production, and increased apoptosis; (6) Spatial and cellular heterogeneity with infiltrative margins, hindering uniform virus distribution and immune activation. Arrows indicate an increase (↑) or decrease (↓) in the respective process.

Although oHSV has clear biological advantages as a therapeutic platform, its efficacy in GBM is ultimately determined not only by viral design but also by the tumor ecosystem into which it is introduced. GBM is characterized by a highly immunosuppressive and spatially heterogeneous microenvironment that limits viral spread, constrains immune activation, and promotes therapeutic resistance. As a result, the discrepancy between promising preclinical activity and variable clinical benefit cannot be understood solely in terms of viral engineering; it must also be interpreted through the lens of tumor–host interactions within the GBM microenvironment. Recent reviews and clinical-translational studies have consistently identified microenvironmental restriction as one of the principal barriers to successful oncolytic virotherapy in GBM [5]. These biological and physical barriers are summarized schematically in Figure 3.

### 4.1. Limited Intratumoral Viral Distribution

One of the most immediate obstacles facing oHSV after administration is inadequate intratumoral distribution. Even when the virus is delivered directly into the tumor or resection cavity, its ability to diffuse throughout GBM tissue is restricted by the dense and irregular extracellular matrix, elevated interstitial pressure, focal necrosis, and the infiltrative growth pattern of GBM cells. These features create a situation in which viral infection may remain confined to limited local regions while invasive tumor cells beyond the injection zone escape exposure. Because GBM recurrence often arises from dispersed infiltrating cells rather than the central tumor mass alone, incomplete viral coverage can substantially reduce therapeutic impact. This issue has been repeatedly highlighted as a central translational limitation of oncolytic therapy in glioma [5,21].

### 4.2. Innate Antiviral Immunity Restricts Viral Replication

A second major barrier is the host innate antiviral response. Although defective antiviral signaling in tumor cells can create opportunities for selective viral replication, GBM tissue is not uniformly permissive. Type I interferon signaling, PKR-mediated translational shutdown, and other intrinsic antiviral programs can suppress viral protein synthesis and replication before the infection has spread sufficiently to generate a meaningful oncolytic effect. In practice, this means that the same host defense mechanisms that protect normal tissue from viral injury may also curtail therapeutic efficacy inside the tumor. Newer vectors such as C134 and transcriptionally targeted constructs were designed partly to address this problem, underscoring how central antiviral restriction has become in oHSV development [26,27].

### 4.3. Macrophages and Microglia as a Double-Edged Component of the TME

Glioma-associated macrophages and microglia represent one of the most important cellular barriers to oHSV therapy. These myeloid populations constitute a major fraction of the GBM microenvironment and often adopt phenotypes that support tumor growth, invasion, angiogenesis, and immune suppression. In the context of virotherapy, they also exert antiviral functions that can rapidly clear infected cells or limit viral propagation. Experimental work has shown that macrophage- and microglia-derived inflammatory mediators, including TNF-α, can suppress oHSV replication in GBM models, illustrating the paradox at the center of viro-immunotherapy: an immune response is needed for durable antitumor control, but an early or dominant antiviral myeloid response may terminate viral replication too soon [28]. This tension is especially relevant in GBM, where myeloid cells are abundant and often dominate the local immune landscape.

From a functional perspective, the contribution of macrophages and microglia to oHSV therapy cannot be fully reduced to the classical M1/M2 dichotomy, but this framework remains useful for understanding their distinct influences. M1-like myeloid cells are generally associated with pro-inflammatory cytokine release, antiviral defense, antigen presentation, and early restriction of viral replication. By contrast, M2-like macrophages are more commonly linked to tissue repair, immune suppression, tumor invasion, and support of GBM progression. In the setting of oHSV therapy, this creates a dual challenge: an M1-skewed response may prematurely suppress viral amplification, whereas an M2-dominant microenvironment may preserve tumor-supportive immune suppression and weaken durable antitumor immunity. Thus, effective oHSV therapy may require not simply greater inflammation, but better-timed and better-balanced reprogramming of the myeloid compartment.

### 4.4. Profound T-Cell Dysfunction and Immunologic “Coldness”

In addition to myeloid dominance, GBM is characterized by weak and often dysfunctional adaptive immunity. T-cell infiltration is variable, effector activity is frequently impaired, and tumor-reactive responses are constrained by local inhibitory signaling and systemic immunosuppressive influences. This immunologically “cold” state helps explain why oHSV, despite its potential to release tumor antigens and danger signals, does not always generate a sufficiently durable adaptive response on its own. The Nature study of CAN-3110 is important in this regard because it provided human evidence that intratumoral oHSV can enhance anticancer immune activation even within immunosuppressive recurrent GBM, but it also implicitly highlighted that such immune conversion is not universal and likely depends on both tumor context and host factors [19].

### 4.5. Inter- and Intratumoral Heterogeneity

GBM heterogeneity further complicates oHSV therapy at multiple levels. Different tumor regions may vary in proliferative status, antiviral competence, metabolic condition, stem-like phenotype, and immune composition. Likewise, different patients may harbor tumors with distinct transcriptional states and microenvironmental architectures. This heterogeneity means that susceptibility to viral infection and the downstream immune consequences of virotherapy are unlikely to be uniform [21]. Some regions may support efficient replication, whereas others may rapidly abort infection or fail to convert viral lysis into productive antitumor immunity. Heterogeneity therefore acts not only as a general mechanism of treatment resistance, but also as a specific obstacle to consistent oHSV performance across patients and within individual tumors.

### 4.6. The Blood–Brain and Blood–Tumor Barriers

Although most clinical oHSV strategies in GBM rely on local rather than systemic delivery, the blood–brain barrier and blood–tumor barrier still matter. They influence immune-cell trafficking, cytokine gradients, vascular permeability, and the distribution of systemically administered combination agents. More broadly, the compartmentalized CNS environment shapes how antiviral and antitumor immune responses develop after viral administration. This helps explain why strategies that are successful in extracranial tumors do not always translate directly to GBM. Reviews of malignant glioma oHSV therapy increasingly discuss delivery and barrier biology together, reflecting the reality that successful virotherapy in the brain is as much an issue of microenvironmental access as of viral design [27,29,30,31].

### 4.7. Microenvironmental Barriers Help Explain the Need for Combination Therapy

Taken together, these barriers show that the limitations of oHSV in GBM are not simply due to insufficient lytic power [5]. Rather, they arise from a mismatch between viral potential and the suppressive, heterogeneous, and spatially restrictive ecology of GBM. This is why modern oHSV development increasingly emphasizes immune-arming, transcriptional targeting, and rational combinations with radiotherapy or checkpoint blockade [31,32]. In effect, the microenvironment has become the main therapeutic battlefield: success will depend not only on infecting tumor cells, but also on reshaping the local conditions that determine whether viral infection can propagate, persist long enough to matter, and culminate in durable antitumor immunity.

## 5. Preclinical and Clinical Progress of oHSV in GBM

The development of oHSV therapy for GBM has proceeded through a translational trajectory in which preclinical innovation repeatedly informed clinical design, while early-phase clinical trials in turn exposed the biological and logistical constraints that now shape next-generation vectors. Broadly, this progress can be understood in two interconnected phases: first, the establishment of preclinical proof-of-concept that genetically attenuated or engineered HSV-1 can selectively target glioma while preserving CNS safety; and second, the gradual emergence of human data demonstrating safety, feasibility, immunologic activity, and, in selected settings, preliminary signals of clinical benefit. Although durable disease control remains inconsistent, the accumulated evidence has clearly established oHSV as one of the most mature and clinically advanced oncolytic platforms in neuro-oncology.

### 5.1. Preclinical Progress

Early preclinical studies were essential in demonstrating that HSV-1 could be genetically attenuated for intracranial use while retaining antitumor activity in malignant glioma. The development of first-generation constructs such as G207 showed that dual *γ34.5* deletion combined with *ICP6* disruption could markedly reduce neurovirulence yet preserve tumor-selective replication in proliferating glioma cells [9,23]. These findings established the central translational principle that oHSV could be rendered sufficiently safe for brain application without being reduced to a biologically inert vector. Subsequent safety work in non-human primates further strengthened the platform by showing that intracerebral administration of attenuated replication-competent HSV could be tolerated under carefully controlled conditions [25].

As the field matured, preclinical development shifted from basic attenuation toward improving replication, spread, and immune activation within the hostile GBM microenvironment. This led to second-generation strategies such as C134, which incorporates HCMV *IRS1* to enhance late viral protein synthesis under antiviral stress, and M032, which adds human IL-12 to combine direct oncolysis with local immune stimulation [17,26]. In parallel, transcriptionally retargeted constructs such as rQNestin34.5v.2 were developed to restore critical viral functions selectively in *nestin*-expressing glioma cells, thereby addressing the potency limitations of highly attenuated first-generation vectors. Collectively, these studies moved the field beyond the question of whether HSV could be used in the brain at all, toward the more nuanced question of how to optimize the balance among safety, replication competence, tumor specificity, and immunologic remodeling.

Preclinical work has also consistently suggested that oHSV is unlikely to achieve maximal benefit as a purely stand-alone cytolytic agent [21]. Multiple studies and reviews have emphasized that the most meaningful advances arise when viral therapy is integrated with broader biological strategies, including radiotherapy, immune checkpoint blockade, or microenvironment-directed modulation. This insight is important because it reframes preclinical success not as simple tumor lysis, but as the capacity of oHSV to act as a multifunctional therapeutic backbone capable of both direct tumor killing and immune conversion.

### 5.2. Early Clinical Translation: G207 as a Foundational Milestone

The earliest phase I clinical trials of G207 marked a turning point in the translation of oHSV for malignant glioma. In the 2000 Gene Therapy study, intracerebral administration of G207 in patients with recurrent malignant glioma demonstrated that replication-competent genetically engineered HSV-1 could be delivered into human brain tumors with an acceptable safety profile, without evidence of HSV encephalitis or dose-limiting CNS toxicity [13]. Although the study was not designed to prove efficacy, it established the clinical feasibility of oHSV-based therapy and provided the first direct evidence that this platform could move beyond laboratory investigation into human neuro-oncology.

Subsequent work expanded on this foundation. A later phase Ib trial evaluated G207 administered both before and after tumor resection in recurrent GBM, again reinforcing feasibility and safety while also providing a practical model for perioperative viral delivery [15]. This approach aligned well with neurosurgical management of recurrent glioma, where direct cavity-based or intratumoral administration can be integrated into operative workflows. Together, these early G207 studies did not resolve the efficacy question, but they removed what had initially been the greatest barrier to the field: the concern that intracranial use of replication-competent HSV would be clinically unacceptable.

The translational importance of G207 was further strengthened by a phase I trial in which the virus was combined with radiation in recurrent GBM [16]. This 2014 Molecular Therapy study demonstrated that G207 plus a single low dose of radiation remained safe and was associated with radiographic responses in some patients, supporting the idea that oHSV may work more effectively in a multimodal context than as monotherapy [16]. Even though the sample size was small and the study was not powered for definitive survival conclusions, it provided one of the earliest clinical indications that rational combination therapy might improve the biological impact of oHSV in GBM. Importantly, these early G207 trials involved relatively small cohorts and were primarily designed to assess safety and feasibility rather than definitive survival benefit, which should be considered when interpreting the observed radiographic and clinical signals.

### 5.3. Immune-Armed and Next-Generation Clinical Strategies

Following the establishment of safety and feasibility with first-generation vectors, clinical development began to shift toward immune-armed and biologically more sophisticated vectors. M032 represents an important example of this transition. The phase I study design published in 2016 described a dose-escalation trial of an IL-12-expressing *γ34.5*-deleted HSV-1 for recurrent or progressive malignant glioma, explicitly framing the virus not only as an oncolytic agent but also as a local immune activator [17]. Even before mature outcome data became available, the design itself reflected a conceptual shift in the field: therapeutic engineering was now being used to integrate virotherapy with immunomodulation at the point of delivery.

A parallel line of development sought to recover viral potency more selectively rather than simply add immune payloads. This logic culminated in *nestin*-targeted constructs such as CAN-3110, which re-express *γ34.5* under control of a tumor-associated regulatory program. The 2023 Nature report on the first-in-human phase I trial of CAN-3110 in recurrent GBM is especially important because it moved the field beyond simple safety confirmation [19]. The study reported treatment in 41 patients and linked intratumoral oHSV administration to immunologic activation, while also identifying associations between biological response patterns and survival. These findings do not yet establish oHSV as a standard therapy, but they provide some of the strongest clinical evidence to date that appropriately engineered HSV can produce measurable immunobiological effects in human recurrent GBM. These findings provide strong translational support for biologic activity, although larger and more comparative studies are still needed to define the magnitude and reproducibility of clinical benefit.

### 5.4. G47Δ/Teserpaturev and the Significance of Regulatory Progress

An especially notable milestone in the clinical history of oHSV for GBM is G47Δ (teserpaturev), a triple-mutated HSV-1 derivative that achieved conditional and time-limited approval for malignant glioma in Japan in 2021 [33,34,35]. This approval was supported by a phase II study in residual or recurrent GBM published in *Nature Medicine* in 2022 [33]. In that trial, repeated intratumoral administration of G47Δ in 19 patients produced encouraging survival outcomes, including a 1-year survival rate of 84.2%. Although the study was single-arm and relatively small, its regulatory and clinical significance is substantial: it demonstrated that an oHSV platform could progress beyond exploratory early-phase testing to formal approval in a major jurisdiction for malignant glioma. For the field as a whole, G47Δ serves as proof that oHSV-based therapy is not merely an experimental concept, but a clinically actionable modality under defined regulatory conditions [34]. Although the survival findings were encouraging, the interpretation remains constrained by the single-arm design, limited sample size, and the absence of a randomized control group.

### 5.5. What the Clinical Record Has Actually Shown

Taken together, the clinical literature supports three conclusions [9]. First, oHSV therapy for GBM has shown a consistently acceptable safety profile across multiple engineered platforms when administered locally under controlled conditions. Second, there is growing evidence that oHSV can induce biologically meaningful immune remodeling in at least a subset of patients, particularly with next-generation constructs such as CAN-3110 and immune-oriented designs such as M032. Third, while signals of efficacy have been observed, especially in selected trials and in the G47Δ experience, durable benefit remains variable and the field has not yet reached a point where clinical outcomes are predictable across patients. These mixed but steadily improving results help explain why current development is increasingly focused on combination regimens, biomarker-guided selection, and iterative viral engineering rather than on simple expansion of first-generation monotherapy models [21,36].

### 5.6. Translational Implications

The preclinical and clinical trajectory of oHSV in GBM suggests that the field has now moved beyond proof-of-concept. The main challenge is no longer whether oHSV can be delivered safely into brain tumors, but how to maximize its biological impact in a disease defined by heterogeneity, antiviral restriction, and profound immune suppression. In this context, future progress will likely depend on three linked strategies: improving patient selection, integrating oHSV with rational combination therapies, and designing response assessment frameworks that capture both delayed immune benefit and nonconventional imaging changes. These issues form the basis for the next stage of oHSV development in GBM.

A comparison across representative studies suggests that more favorable outcomes have generally been associated with one or more of the following features (Table 2): improved replication competence beyond first-generation attenuation alone, immune-arming strategies that enhance microenvironmental conversion, and treatment settings that better integrate local viral therapy with broader antitumor mechanisms. By contrast, less impressive or more ambiguous outcomes have often been observed when viral spread remained spatially restricted, attenuation substantially reduced replicative fitness, or study designs were primarily optimized for safety rather than biological stratification. Although such comparisons should be interpreted cautiously because of major differences in models and endpoints, they provide useful guidance for future vector development and trial design.

### 5.7. Lessons from Negative or Inconclusive Studies

Not all oHSV studies in GBM have produced clear efficacy signals, and these less successful or inconclusive experiences have been highly informative for the field. In several early clinical studies, including trials involving first-generation attenuated vectors, safety and feasibility were convincingly demonstrated, yet durable survival benefit remained limited or difficult to interpret. This does not necessarily imply biological inactivity. Rather, it reflects several recurring constraints, including small sample size, heavily pretreated recurrent-disease populations, modest replication capacity of highly attenuated vectors, and the absence of biomarker-guided patient selection.

These studies also clarified an important translational lesson: safety alone is not sufficient for meaningful efficacy in GBM. Excessive attenuation may reduce neurovirulence but can also compromise intratumoral replication and spread, particularly in the highly restrictive glioblastoma microenvironment. In addition, early-phase study designs were often not intended to distinguish transient radiographic or immunologic changes from durable clinical benefit. Taken together, these less favorable or inconclusive outcomes have played an important role in redirecting the field toward better-balanced vector design, immune-arming strategies, and more mechanism-oriented combination approaches. Similar lessons can also be drawn from early HSV1716 experience, in which biological feasibility was demonstrated but the overall translational impact remained constrained by limited efficacy signals and the broader challenges of early-stage glioma virotherapy development [37].

## 6. Rational Combination Strategies for oHSV in GBM

The accumulated preclinical and clinical literature suggests that oHSV is unlikely to reach its full therapeutic potential in GBM as a stand-alone modality. This is not because the platform lacks biological activity, but because the major barriers to success in GBM are multifactorial: limited viral spread, antiviral innate immunity, myeloid-dominant immune suppression, adaptive T-cell dysfunction, and marked spatial heterogeneity. For that reason, current development has increasingly shifted toward rational combination strategies designed either to enhance viral replication, amplify immune conversion, or improve control of residual infiltrative disease after local treatment. Recent reviews now frame combination therapy not as an optional add-on, but as the central path forward for oHSV in GBM.

### 6.1. Combination with Radiotherapy

Radiotherapy is one of the most natural partners for oHSV in GBM. From a biological standpoint, radiation can increase tumor cell stress, alter antiviral and DNA-damage response pathways, and enhance immunogenicity, thereby creating a more permissive setting for viral activity. At the same time, oHSV may complement radiotherapy by extending tumor cell killing beyond the immediate radiation field and by promoting inflammatory remodeling of the tumor microenvironment. This rationale has been supported clinically by the phase I study of G207 plus radiation in recurrent GBM, which demonstrated that the combination was feasible and safe and provided early evidence that multimodal treatment may yield stronger biological effects than viral monotherapy alone [16,38]. More recent preclinical reviews continue to identify radiation as one of the most credible combination backbones for malignant glioma virotherapy [31].

### 6.2. Combination with Immune Checkpoint Blockade

The combination of oHSV with immune checkpoint blockade is especially attractive because the two modalities address complementary limitations. GBM is typically a poorly inflamed tumor with limited baseline responsiveness to PD-1/PD-L1-directed therapy, while oHSV has the potential to increase antigen release, inflammatory signaling, and immune-cell recruitment within the tumor. In theory, viral therapy can help convert an immunologically “cold” tumor into a more checkpoint-responsive state, while checkpoint blockade may help sustain and expand the adaptive antitumor responses initiated by viral infection. This concept has become a recurring theme in both GBM-focused and broader OV-immunotherapy reviews, and it is now one of the most actively discussed translational directions for oHSV [7,39]. Still, the combination is not automatically synergistic; its success likely depends on timing, viral design, and the balance between antiviral clearance and antitumor immunity.

### 6.3. Immune-Armed oHSV as an Intrinsic Combination Strategy

One of the most important conceptual advances in the field is that combination therapy does not always require two separate drugs. As detailed in Section 3.3, immune-armed oHSV constructs effectively embed combinatorial biology within a single vector by coupling direct oncolysis with localized cytokine or immune-modulatory delivery. The aforementioned IL-12-expressing oHSV is a leading example [17]. Recent preclinical work has shown that unarmed oHSV may fail to produce durable benefit in immunocompetent glioma models, whereas IL-12-armed constructs can promote macrophage remodeling, cytotoxic T-cell accumulation, and more sustained survival benefit [17]. This supports a broader view of oHSV not merely as a replicating agent, but as a programmable immunotherapeutic platform capable of locally delivering the second signal that GBM often lacks.

### 6.4. Combination with Other Immunomodulatory Strategies

Beyond checkpoint inhibitors and cytokine arming, oHSV is also being positioned within a broader immunomodulatory framework. Recent reviews of OV-based combination therapy describe strategies involving cytokine support, myeloid reprogramming, and enhancement of T-cell recruitment or expansion [14,21]. In GBM, these approaches are particularly relevant because the dominant barrier is often not simple tumor-cell resistance, but the persistence of an immunosuppressive microenvironment that aborts durable immune control. This is why newer constructs and combinations increasingly target not only tumor lysis, but also the quality of the immune response generated after infection. The field is moving from “virus plus immunity” to deliberately engineered viro-immunotherapy ecosystems.

### 6.5. Perioperative and Post-Resection Strategies

Because GBM treatment is fundamentally anchored in surgery, perioperative use of oHSV is particularly relevant. Viral administration into the resection cavity or residual tumor margin can exploit a window in which tumor burden is reduced, direct access is available, and locoregional therapy can be concentrated at the site where recurrence is most likely to emerge [15]. More broadly, recent virotherapy reviews note that surgery itself may create a biologically important interval in which local immunologic and stromal conditions are altered, making post-resection treatment especially strategic [9]. Experimental post-resection local-delivery studies outside strict oHSV-only settings also support the idea that locoregional immune stimulation after surgery can improve residual disease control. For GBM, this reinforces the logic that oHSV may be most effective when embedded in the perioperative treatment sequence rather than used only as a late salvage monotherapy.

### 6.6. Cell-Based Delivery and Other Delivery-Enhancing Combinations

A recurring limitation of oHSV in GBM is restricted distribution after local injection. This has motivated interest in delivery-enhancing strategies, including carrier-cell approaches such as mesenchymal stromal/stem cells. Recent reviews of cell-carrier platforms note that carrier-based delivery may help protect virus from premature clearance and improve distribution within infiltrative brain tumors, although systemic administration still faces substantial challenges [9,14]. While these approaches remain less clinically mature than direct intratumoral delivery, they are relevant to the future combination landscape because they target one of the most persistent weaknesses of oHSV: inadequate reach into dispersed residual disease.

### 6.7. What Combination Strategies Are Really Trying to Solve

Viewed together, these combinations are addressing three core problems. The first is insufficient potency, for which radiation and conditional engineering may increase effective tumor killing. The second is inadequate immune conversion, for which cytokine arming and checkpoint blockade are intended to amplify adaptive responses. The third is limited spatial coverage, for which perioperative delivery and carrier-based strategies attempt to improve access to residual infiltrative tumor. This framework is useful because it shifts the discussion away from simply listing combinations and toward identifying the precise biological bottleneck each partner is meant to overcome. In GBM, that level of mechanistic alignment is likely to be essential if future trials are to move beyond feasibility and generate reproducible survival benefit.

### 6.8. Optimizing Combinatorial Strategies

The future of oHSV in GBM will almost certainly be combinatorial. Existing evidence supports radiotherapy as a practical backbone, immune checkpoint blockade as a logical but context-dependent partner, immune-armed vectors as a powerful way to embed synergy into the virus itself, and perioperative local treatment as a strategically important delivery setting. The challenge now is to define which combinations should be prioritized, in which patients, and at what point in the treatment course. That next step will require not only better-designed vectors, but also biomarker-informed clinical trials and response frameworks that can capture delayed and immune-mediated benefit (Table 3).

## 7. Current Challenges and Future Directions

Despite substantial progress in vector design and early-phase clinical translation, oHSV therapy for GBM has not yet achieved broadly reproducible and predictable clinical benefit. The main challenge is no longer conceptual feasibility. Instead, the field now faces a more difficult translational problem: how to convert a biologically compelling therapy into a durable and scalable treatment strategy for a highly heterogeneous, locally invasive, and immunologically restrictive disease. Recent reviews consistently frame the next phase of development around several linked priorities, including better patient selection, improved delivery and spatial coverage, more informative response assessment, and biomarker-guided rational combination strategies.

### 7.1. Patient Selection Remains Underdeveloped

One of the most important unresolved issues is patient selection. Current oHSV trials in GBM have shown that not all tumors respond similarly, and emerging clinical data suggest that biological context matters. The CAN-3110 study was especially important because it linked intratumoral oHSV treatment to immunologic activation and survival-associated biological patterns, implying that future benefit may depend on identifying tumors or immune states that are particularly permissive for productive viro-immunotherapy. However, no widely accepted selection framework yet exists for deciding which patients are most likely to benefit from a given oHSV platform. Future progress will likely require integrating tumor molecular features, immune microenvironment characteristics, and perhaps even antiviral-response signatures into trial design rather than relying solely on histologic diagnosis and recurrence status.

### 7.2. Delivery and Spatial Coverage Are Still Major Bottlenecks

A second unresolved challenge is delivery. Most current oHSV approaches in GBM rely on local injection into tumor tissue or the resection cavity, which is logical from a neurosurgical standpoint but does not fully solve the problem of infiltrative residual disease. GBM recurrence often arises from tumor cells extending beyond the immediate treatment field, and limited viral dispersion remains one of the most frequently cited barriers to durable control. Future directions in this area include optimizing locoregional administration strategies, embedding virotherapy more effectively into the perioperative window, and exploring carrier-assisted delivery systems that may improve distribution or shield virus from premature inactivation. None of these approaches has yet fully solved the reach problem, but they directly target one of the most persistent weaknesses in current oHSV therapy.

### 7.3. Balancing Antiviral Immunity and Antitumor Immunity

A defining biological tension in oHSV therapy is that the host immune system is both necessary and potentially counterproductive. Productive viral infection can release tumor antigens and stimulate immune priming, but overly rapid antiviral clearance may terminate replication before sufficient intratumoral spread or immune conversion occurs. This issue is particularly pronounced in GBM, where myeloid-driven innate responses and profound adaptive dysfunction coexist in a way that may restrict viral amplification while still failing to generate durable antitumor immunity. Future vector design will therefore need to become more precise, not simply more potent. The central goal is not maximal inflammation at any cost, but controlled immunologic programming that preserves enough viral activity to matter while steering the downstream response toward effective tumor rejection.

### 7.4. Biomarkers Need to Move from Exploratory to Practical

Biomarker development is likely to be one of the most important determinants of future clinical success. At present, biomarkers in the oHSV-GBM field remain largely exploratory. Yet the broader OV literature increasingly emphasizes predictive and pharmacodynamic markers as essential tools for improving trial efficiency and interpreting heterogenous responses. In GBM, useful biomarker categories may include tumor-cell permissiveness markers, baseline immune-state indicators, signatures of myeloid suppression, viral gene-expression readouts, cytokine changes after treatment, and composite response markers that better reflect delayed immunologic benefit. The field will probably need practical biomarker panels rather than single-marker solutions, because response to oHSV is unlikely to be determined by one variable alone.

### 7.5. Response Assessment Remains Difficult

Another major challenge is how to evaluate treatment response. GBM imaging is already complicated by pseudoprogression and treatment-related change under standard chemoradiation, and these problems become even more difficult when immunologic and inflammatory effects are intentionally induced by virotherapy. Conventional imaging criteria may underestimate delayed benefit or misclassify inflammatory enlargement as tumor progression. This creates a serious translational problem: if response is assessed too early or too simplistically, biologically active therapies may be abandoned before their benefit becomes visible. Future trials of oHSV will need more refined assessment frameworks that combine imaging, clinical course, and biological correlates, rather than relying only on structural MRI changes at fixed early time points.

### 7.6. Combination Therapy Must Become More Selective, Not Just More Complex

Combination therapy is almost certainly the future of oHSV in GBM, but the next generation of studies cannot simply add more agents. The central question is which combination addresses which bottleneck. Radiotherapy may enhance local cytotoxicity and immunogenic stress; checkpoint blockade may help sustain adaptive responses after viral priming; immune-armed vectors may deliver cytokine support directly into the tumor; and delivery-focused strategies may improve spatial reach. The challenge now is prioritization. Future combination trials should be structured around mechanism-based hypotheses and biomarker-defined subgroups rather than broad empiricism. That shift from “promising combinations” to “precision combinations” will likely determine whether the field can move from intriguing signals to reproducible benefit.

### 7.7. From Feasibility to Strategy

The broader trajectory of oHSV in GBM can be understood as a transition through three stages. The first stage asked whether engineered HSV could be safely delivered into brain tumors. The second asked whether it could generate measurable biological and clinical activity in humans. The field is now entering a third stage, in which the goal is strategic optimization: selecting the right patients, designing the right vector for the right tumor context, combining it with the right partner therapy, and evaluating benefit with the right endpoints. The conditional approval of G47Δ in Japan and the human immunobiological signals seen with CAN-3110 have made it clear that oHSV is no longer a speculative platform. The remaining challenge is to define how it should be used most intelligently.

### 7.8. Summary

In future development, the most important advances are likely to come not from a single breakthrough vector, but from convergence: better engineering, better delivery, better biomarkers, better trial design, and better integration with the biology of recurrent GBM. If these elements can be aligned, oHSV may evolve from a promising experimental modality into a more consistently effective component of GBM therapy. At present, the evidence supports cautious optimism: the platform has crossed the threshold of feasibility, but its maturation into standardizable therapy will depend on how successfully the field addresses the translational questions that now define it.

## Figures and Tables

**Table 1:** Representative engineered oHSV platforms for glioblastoma.

Platform	Key Genetic Modifications	Main Engineering Purpose	Main Advantage	Main Limitation	Development Status
G207	Deletion of both *γ34.5* genes; insertional inactivation of *ICP6 (UL39)*	Reduce neurovirulence and improve tumor-selective replication	Landmark safety-oriented platform; established clinical feasibility of intracranial oHSV therapy	Replication potency may be limited because of strong attenuation	Phase I/Phase Ib clinical studies
G47Δ (teserpaturev)	Based on G207 backbone with additional deletion of *α47* and altered *US11* expression	Enhance antitumor activity and antigen presentation while maintaining safety	More potent than earlier first-generation vectors; most clinically advanced approved oHSV platform in malignant glioma	Clinical evidence is still based on relatively small studies	Phase II; conditionally approved in Japan for malignant glioma
M032	*γ34.5*-deleted HSV-1 engineered to express human IL-12	Combine direct oncolysis with local immune stimulation	Represents transition from pure oncolysis to viro-immunotherapy	Clinical efficacy remains under evaluation	Phase I clinical development
C134	*γ34.5*-deleted HSV-1 expressing HCMV *IRS1*	Improve replication under host antiviral restriction while preserving safety	Attempts to restore viral fitness without fully reversing attenuation	Clinical experience is more limited than G207/G47Δ	Advanced preclinical/early clinical translation
CAN-3110	Tumor-selective *γ34.5* expression driven by a *nestin*-related regulatory strategy	Restore replication competence preferentially in glioma cells	Precision design balancing safety and potency; associated with immunologic activation in recurrent high-grade glioma	Benefit may depend on tumor context and patient selection	Phase I clinical study

Abbreviations: oHSV, oncolytic herpes simplex virus; HSV-1, herpes simplex virus type 1; HCMV, human cytomegalovirus; IL-12, interleukin-12; *IRS1*, infected cell ribosome shutoff repressor 1.

**Table 2:** Key preclinical and clinical studies of oHSV in glioblastoma.

Study Type	Platform/Study	Model or Population	Dose/Schedule	Delivery Route/Setting	Observation/Study Endpoint	Main Findings	Key Limitation/Implication
Preclinical	G207	Malignant glioma models; later supported by non-human primate safety work	Variable by model	Intratumoral/intracerebral experimental delivery	Safety, tumor-selective replication, antitumor activity	Established the foundational principle that dual *γ34.5* deletion plus *ICP6* disruption could reduce neurovirulence while preserving antitumor activity in glioma cells	Strong proof-of-concept, but first-generation attenuation may restrict replication/spread in hostile GBM microenvironments
Preclinical	C134	Murine glioma models and non-human primates	Experimental dosing; platform designed to improve replication under antiviral stress	Intracerebral/intratumoral	Replication competence, safety, biodistribution, antitumor effect	Incorporation of HCMV *IRS1* improved late viral protein synthesis and partially restored fitness relative to highly attenuated vectors	Illustrates the trade-off between safety and potency; translational relevance lies in selective recovery of replication rather than simple attenuation
Preclinical	M032	Immunocompetent glioma models; translational preclinical studies	Engineered IL-12-expressing oHSV; experimental repeated-dose frameworks in preclinical work	Intratumoral/intracerebral	Immune activation, survival, safety	Demonstrated that immune arming can convert oHSV from a mainly lytic platform into a local immunomodulatory therapy with stronger antitumor immune effects	Supports the idea that direct oncolysis alone is insufficient and that immune remodeling is critical in GBM
Preclinical	rQNestin34.5v.2	*Nestin*-positive glioma models	Experimental dosing varies by study	Intratumoral	Tumor-selective replication, efficacy, safety	Conditional re-expression of *γ34.5* under a *nestin*-associated program improved tumor-selective competence while preserving the rationale for CNS safety	Represents the shift from static attenuation to conditional tumor-restricted potency restoration
Clinical	G207 phase I (Markert et al., 2000; USA [13])	Recurrent malignant glioma	Dose-escalation; single-agent oHSV	Intracerebral inoculation	Primary: safety and feasibility	Demonstrated that replication-competent genetically engineered HSV-1 could be administered into human brain tumors with acceptable safety and without HSV encephalitis	Foundational safety trial, but not designed to establish efficacy or survival benefit
Clinical	G207 phase Ib (Markert et al., 2009; USA [15])	Recurrent GBM/malignant glioma undergoing resection	Pre- and post-resection administration	Intratumoral and peri-resection cavity setting	Feasibility, perioperative delivery, safety	Showed that perioperative viral delivery could be integrated into neurosurgical workflows and remained feasible and safe	Reinforced delivery feasibility, but efficacy interpretation remained limited by cohort size and early-phase design
Clinical	G207 + radiation phase I (Markert et al., 2014; USA [16])	Recurrent GBM	G207 combined with a single low dose of radiation	Local viral administration plus radiotherapy	Safety, radiographic response, feasibility	Combination was safe and associated with radiographic responses in some patients, supporting multimodal development	Encouraging signal, but sample size was small and conclusions regarding survival remained preliminary
Clinical	M032 phase I design (Patel et al., 2016; USA [17])	Recurrent/progressive GBM, anaplastic astrocytoma, or gliosarcoma	Dose-escalation; IL-12-expressing oHSV	Intratumoral	Safety and translational feasibility	Marked a conceptual transition from simple oncolysis toward immune-armed clinical virotherapy	Important for design logic, but mature efficacy conclusions require completed outcome data
Clinical	CAN-3110 phase I (Ling et al., 2023; USA [19])	Recurrent GBM	Intratumoral treatment; schedule per phase I protocol	Intratumoral	Safety, immunologic activation, survival associations	Linked oHSV treatment to intratumoral immune activation and survival-associated biological patterns, providing strong human translational evidence	Important biologic signal, but broader efficacy validation still requires larger comparative studies
Clinical	G47Δ/teserpaturev phase II (Todo et al., 2022; Japan [33])	Residual or recurrent GBM	Repeated intratumoral administration	Intratumoral	1-year survival and clinical outcomes	Produced encouraging survival outcomes, including a reported 1-year survival rate of 84.2%, and supported conditional/time-limited approval in Japan	Major milestone for the field, but interpretation remains constrained by single-arm design and modest sample size

Abbreviations: oHSV: Oncolytic Herpes Simplex Virus; G207: a genetically engineered, replication-competent, conditionally attenuated HSV-1 strain; M032: a genetically engineered oHSV-1 strain expressing human interleukin-12 (IL-12); C134: a second-generation oHSV-1 strain designed with enhanced intratumoral replication fitness; CAN-3110: an HSV-1-based oncolytic viral therapy whose replication is regulated by a *nestin* promoter; G47Δ (teserpaturev): a third-generation, genetically modified oHSV-1 strain based on G207, with teserpaturev as its International Nonproprietary Name; GBM: Glioblastoma; IL-12: Interleukin-12; HSV-1: Herpes Simplex Virus type 1; HCMV *IRS1*: Human Cytomegalovirus Internal Repeat Short Segment 1; *γ34.5*: the HSV-1 neurovirulence gene ICP34.5; ICP6: the HSV-1 ribonucleotide reductase large subunit (gene *ICP6* or *UL39*); *nestin*: a neural progenitor and glioma cell marker.

**Table 3:** Rational combination strategies of oHSV in glioblastoma.

Combination Strategy	Main Rationale	Expected Benefit	Current Support	Main Unresolved Issue
oHSV + radiotherapy	Radiation may increase tumor stress, enhance immunogenicity, and improve the biological context for viral activity	Stronger local tumor control and potentially enhanced viral efficacy	Early clinical and preclinical support	Optimal dose, timing, and sequencing remain unclear
oHSV + immune checkpoint blockade	oHSV may increase antigen release and immune-cell recruitment, potentially converting “cold” tumors into more responsive lesions	More durable adaptive antitumor immunity	Strong mechanistic rationale; growing preclinical/translational interest	Benefit likely depends on timing, patient selection, and immune context
Immune-armed oHSV (for example, IL-12-expressing vectors)	Combines direct oncolysis with local cytokine-mediated immune activation	Enhanced immune remodeling within the tumor microenvironment	Supported by translational and preclinical studies; clinical development underway	How to maximize immune benefit without excessive toxicity remains uncertain
oHSV + perioperative/local delivery strategy	Surgery provides direct access to the resection cavity and residual tumor margin	Better local drug delivery and improved integration with standard neurosurgical care	Clinically practical and supported by prior local-delivery studies	Limited reach to infiltrative tumor cells beyond the treatment field
oHSV + cell-carrier or delivery-enhancing approaches	Carrier systems may improve viral distribution and reduce premature inactivation	Better spatial coverage and possibly improved access to dispersed residual disease	Mainly preclinical or early translational support	Clinical feasibility and reproducibility remain to be established

Abbreviations: oHSV: Oncolytic Herpes Simplex Virus; IL-12: Interleukin-12.

## Data Availability

No new data were created or analyzed in this study. Data sharing is not applicable to this article.
